# Endovascular stenting for idiopathic intracranial hypertension with venous sinus stenosis

**DOI:** 10.1002/brb3.1279

**Published:** 2019-04-04

**Authors:** Xinfeng Liu, Hai Di, Jun Wang, Xiangyu Cao, Zhihua Du, Rongju Zhang, Shengyuan Yu, Baomin Li

**Affiliations:** ^1^ Department of Neurology Chinese PLA General Hospital Beijing China

**Keywords:** body mass index, Idiopathic intracranial hypertension, stenting, venous sinus stenosis, vision outcome

## Abstract

**Objectives:**

Idiopathic intracranial hypertension (IIH) is characterized by elevated intracranial pressure of unknown etiology and venous sinus stenting may be an optional treatment. We aimed to evaluate the effects of venous sinus stenting on visual function, intracranial pressure, and trans‐stenotic pressure gradient of the patients with IIH and to determine effects of baseline BMI or weight changes on subjective vision outcome and intracranial pressure.

**Methods:**

From July 2009 to Aug 2016, 88 eligible patients with IIH and venous sinus stenosis who underwent stenting were retrospectively studied.

**Results:**

In this study, 67 women and 21 men were included with an average age of 39.01 (18–60) years. The average BMI was 26.75 kg/m^2^. Here, 66 (75.9%) patients had papilledema, 39 had impaired vision before stenting; 57 patients were followed‐up, 48 (84.2%) showed significant subjective improvement or recovery in visual acuity, 4 (7.0%) patients reported no significant change in visual functions, and 5 (8.8%) suffered permanent vision loss. The cerebrospinal fluid opening pressure and trans‐stenotic pressure gradient were significantly decreased postoperatively. Baseline BMI was associated with pre‐and postoperative trans‐stenotic pressure gradients, as well as changes in cerebrospinal fluid opening pressure. However, baseline BMI and body weight changes during follow‐up were not necessarily associated with subjective visual outcomes after stenting. Stenting efficacy was limited in patients with severe preoperative optic symptoms.

**Conclusions:**

Venous sinus stenting represented an effective treatment for resolving visual dysfunction and intracranial pressure associated with venous sinus stenosis. BMI seemed to be associated with intracranial pressure but not subjective visual outcomes after stenting.

## 1 INTRODUCTION

Idiopathic intracranial hypertension (IIH), also known as primary pseudotumor cerebri, is a complicated syndrome that includes elevated intracranial pressure of unidentified etiology (Sundholm et al., 2017). IIH generally manifests with debilitating headaches, nausea, vision loss, impaired visual fields, diplopia, and pulse–synchronous tinnitus, and it primarily affects overweight women of childbearing age at a rate of 12–28 per 100,000 persons annually (Chagot et al., 2017). Permanent vision loss may affect up to 30% of patients due to IIH‐associated chronic papilledema (Andrews, Liu, & Ko, 2014; Liu et al., 2017). Standard medical treatment includes weight loss, administration of acetazolamide and diuretics, and repeated high‐volume lumbar punctures, followed by optic nerve sheath fenestration or cerebrospinal fluid (CSF) shunting to alleviate secondary symptoms in patients with refractory IIH or progressive visual loss (Satti, Leishangthem, & Chaudry, 2015). However, these treatment modalities do not target the primary pathology. Venous sinus stenosis is an important contributor to IIH, as more than 90% of patients with IIH have complications with venous sinus stenosis (Esfahani et al., 2015). Growing evidence have supported that venous sinus stenting can treat these cases of IIH, because it suppresses stenosis and reduces the pressure gradient across the stenotic segment, which in turn reduces intracranial pressure. However, the efficiency of venous sinus stenting should be evaluated in more clinical centers and geographical populations Moreover, whether intracranial pressure can be controlled within a reasonable range after stenting is still an open question. It is not known whether preoperative clinical parameters, such as BMI, trans‐stenotic pressure gradient, and lumbar puncture pressure are associated visual outcomes. Therefore, our retrospective study focused on the venous sinus stenting for patients with IIH.

## 2 METHODS

### 2.1 Patients

We retrospectively collected and analyzed clinical data from 88 patients who were admitted to our department for symptoms or signs of IIH and who underwent venous sinus stenting for venous sinus stenosis from July 2009 to Aug 2016. IIH diagnosis was based on the modified Dandy's criteria, including frequent intractable headache, persistent or progressive visual dysfunction, pulse–synchronous tinnitus, high intracranial pressure without ventricular enlargement or intracranial mass on imaging, and normal CSF constituents. Magnetic resonance imaging (MRI) of the participants’ brains was performed to exclude other causes of IIH, such as a mass lesion, hydrocephalus, or venous sinus thrombosis. A time‐of‐flight venography was used to assess stenosis of the lateral sinus.

Patients were considered for venous sinus stent implantation if they had one or more of the following conditions: refractory or intolerant‐to‐standard conservative therapy (e.g., administration of carbonic anhydrase inhibitors, weight loss, repeated lumbar puncture with CSF withdrawal), failed surgical procedure, persistent or progressive papilledema, intractable headaches, significant IIH, or a diagnosis of venous sinus stenosis > 50% with a trans‐stenosis pressure gradient ≥ 7 mmHg. Baseline, intraprocedural, and postoperative clinical data for each recruited patient were collected from medical charts and neuroimaging. This study followed the principles outlined in the Declaration of Helsinki. Institutional review board approval was obtained from Chinese PLA General Hospital.

### 2.2 Ophthalmic assessment and lumbar puncture for pressure examination

Detailed ophthalmologic examinations were performed on presentation, before stenting and in the follow‐up. Funduscopic examinations, visual acuity testing, and visual field testing were conducted to evaluate patients’ ophthalmological function. Funduscopic examination identified optic pathologic changes, such as papilledema, optic disc and retinal edema or hemorrhage, and blurred papillary boundary. Visual fields were assessed by manual kinetic Goldman perimetry. Visual acuity was measured using an international vision chart. Optic atrophy was assessed according to clinical symptoms and funduscopic examination and confirmed by visual electrophysiology. Optic tests were performed prior to stent implantation to establish diagnosis and to determine the degree of optic nerve impairment. Lumbar punctures at L3–L4 was performed within one week prior to and one week after the stenting procedure to determine the cerebrospinal fluid opening pressure.

### 2.3 Venous sinus stenting protocol and imaging

A routine anti‐platelet therapy including a daily loading dose of 75 mg of clopidogrel and daily dose of 300 mg of aspirin were prescribed about 5 days prior the stent implantation. For patients with a history of arterial or deep venous thrombosis‐associated disease, an anticoagulated heparin combined with a single antiplatelet therapy was given before the procedure. All stenting procedures were performed under general anesthesia. Arterial femoral access was obtained, preprocedural high‐solution digital subtraction angiography (DSA) was performed to exclude vascular malformation or fistula and to identify general features of the stenosis, including location and degree of the stenosis, the presence of collateral vascular dilatation, arteriovenous circulation time, and filling duration. Meanwhile, venous femoral access was obtained, and a diagnostic venography was performed to further observe the morphological features of the stenosis. Intravenous heparin was maintained based on individual body weight. A guide catheter was advanced into the internal jugular vein ipsilateral to the stenosis and directed into the lateral sinus. A manometer was connected to guide the microcatheter and to determine the intravenous pressure in various venous sinus, ipsilateral, and contralateral jugular veins and in the stenotic segment. The trans‐stenotic pressure gradient was defined as the difference in pressure between the distal and proximal stenotic segments. Pressure was assessed and recorded before and after the procedure. Afterwards, a stent of appropriate size was deployed across the stenosis with the assistance of a guide wire. Angiography or venography was performed once more after releasing the stent to observe the improvement of blood flow in the lesioned sinus and determine if a balloon dilatation could be considered as necessary.

### 2.4 Antiplatelet and anticoagulation regimen

After the stenting procedure, the patients were given low‐molecular heparin for 3 days. They continued antiplatelet treatment (75 mg of clopidogrel daily and 300 mg of aspirin daily) for 3 months. Then, clopidogrel administration was discontinued and aspirin was reduced to 100 mg per day for another 6 months. For patients with thrombophilic disorder, long‐term anticoagulation therapy was recommended.

### 2.5 Postoperative and follow‐up outcomes

The recruited patients in this study were recontacted by telephone in July 2017. They were inquired about the following information: alternation of IIH‐associated symptoms (headaches, dizziness, tinnitus, and visual acuity); having ophthalmic assessment or not and the corresponding results; whether radiographic examinations were performed and the recurrence of restenosis; recent body weight.

### 2.6 Statistical analysis

All statistical analyses were performed using SPSS22.0 (IBM, Chicago, IL). Continuous variables are presented as means ± standard derivation (*SD*) with minimum and maximum values. Categorical variables are expressed as frequency or percentage (%). For continuous variables, a paired or unpaired *t* test was used to compare two groups. For multiple comparisons, a one‐way analysis of variance followed by LSD were performed. A Pearson's chi‐square test or Fisher's exact test was used to compare categorical variables. Pearson's correlation analyses were performed to evaluate the association between each two parameters of BMI, pressure gradient, and lumbar puncture pressure. A multivariate forward stepwise Logistic regression analysis was used to determine independent predictors for clinical outcome after stenting. A *p* value less than 0.05 was considered significant.

## 3 RESULTS

### 3.1 Summary of demographic and ophthalmological characteristics of the patients before stenting

The study comprised 88 eligible patients with an average age of 39.01 (18–60) years, 67 (76.1%) of whom were female. The mean BMI was 26.75 (18.59–40.58) kg/m^2^. Using BMI cut‐off points of 24.5 kg/m^2^ for overweight and 29.5 kg/m^2 ^for obese, 23 (27.06%) patients had normal BMIs, 44 (51.76%) were overweight and 18 (21.18%) patients were obese. Body weight or height data was not available for three patients. Here, 64 patients (72.7%) reported visual impairment, and 51 (58%) reported headaches. Comparable proportions of patients suffered from dizziness and tinnitus (15.9% and 13.6%). Eight patients experienced transient ischemic attack and five cases reported limb weakness. The mean medical duration of the symptoms with multiple presentations was 7.8 (0.4–84.0) months (Table 1).

**Table 1:** Baseline clinical characteristics in patients who have idiopathic intracranial hypertension with venous sinus stenosis

Clinical characteristics	Means ± *SD* (Min‐Max)/*n*%
Gender (female)	67 (76.1%)
Age (years)	39.01 ± 9.73 (18–60)
Body weight (kg)	71.45 ± 10.75 (51.0–102.0)
BMI (kg/m^2^)	26.75 ± 3.79 (18.59–40.58)
Normal <24.5	23 (27.06%)
Overweight 24.5–29.0	44 (51.76%)
Obese >29.0	18 (21.18%)
Clinical duration (Months)	7.8 (0.4–84.0)
Preoperative symptoms	
Dizziness	14 (15.9%)
Visual dysfunction	64 (72.7%)
Tinnitus	12 (13.6%)
Limb weakness	5 (5.7%)
Temporality amaurosis	8 (9.1%)

Since visual impairment is a typical symptom of IIH, we then summarized the ophthalmological changes of the recruited patients. As shown in Table 2, 66 cases (75.9%) of papilledema and 3 cases of fundus hemorrhage were detected by funduscopic examinations before stenting. Out of 39 patients who had impaired vision, which presented most often as decreased visual acuity, 20 (23.0%) had papilledema complications; 7 (8.0%) had severe vision impairment involving one or both eyes before stenting, of which, 3 had residual light perception, three patients could observe only hand or finger movement, and one patient suffered permanent unilateral vision loss. Five patients had optic atrophy. Medial visual acuity was 0.63 (range 0.02 –1.25), and mean defect of visual field was −16.3 dB and −14.5 dB in the left and right eyes of the patients, respectively.

**Table 2:** Preoperative ophthalmological characteristics of the cohort

Preoperative ophthalmological characteristics	*n* (%)
Papilledema	66 (75.9%)
Vision acuity (media and range)	0.63 (0.02–1.25)
Impaired vision with papilledema	20 (23.0%)
Visual field defect (mean ± *SD*)	
Left	−16.3 ± −3.5 dB
Right	−14.5 ± −4.2 dB
Fundus hemorrhage	3 (3.5%)
Diplopia	4 (4.6%)
Optic atrophy	5 (5.7%)
Severe vision impairment	
No or residual light perception	3 (3.5%)
Hand or fingers movement	3 (3.5%)
Unilateral blindness	1 (1.2%)

### 3.2 Stenting procedure

All patients in this study had venous sinus stenosis, located almost exclusively in the lateral venous sinus (98.9%), except for 1 (1.1%) in the sagittal sinus. Among them, 70 stenoses were in the transverse‐sigmoid (T‐S) sinus junction, 13 were in the transverse sinus, and 7 patients had bilateral stenoses at the T‐S junction. A variety of bare metal stents were used. The Precise nitinol carotid stent was used in approximately 80% of patients, followed by Xpert, Wallstent, Solitaire, and PROTÉGÉ stents. Stenting procedures were completed with a technique success rate of 100%. No neurological complications related to stent implantation were reported.

### 3.3 Follow‐up outcomes

In the follow‐up, 57 (64.8%) patients were contacted and 40 of them reported headaches and 55 reported visual impairment prior the stenting. 37 (92.5%) reported that their headaches significantly improved (17/40) or completely resolved (20/40) after the procedure. 28 of them reported significant relief of their symptoms immediately after stent deployment. Symptoms of the remaining patients (9/40) progressively improved over 2–7 weeks after the stent implantation. Besides, 48 (84.2%) patients reported significant improvement or recovery in visual acuity, 4 (7.0%) reported no significant change in visual functions and 5 (8.8%) suffered permanent vision loss, including three single left blindness and two binocular vision loss. All patients who lost their vision had preoperative papilledema, four of them had poor visual function with no or residual light perception before stenting that was complicated by initial optic atrophy. However, 39 patients had further ophthalmic examinations after discharge, all of them had papilledema resolved or almost completely resolved. The media acuity increased to 0.8 (range 0.02–1.25) and mean visual field defects were −9.35 dB and −7.28 dB in the left and right eyes in the follow‐up. Finally, two patients died during follow‐up, one from cerebral infarction and one from the advanced intracranial glioma diagnosed about one year after stenting. No recurrent stenosis was found in these patients.

### 3.4 Trans‐stenosis pressure gradient and cerebrospinal fluid opening pressure after stenting

**Figure 1:**
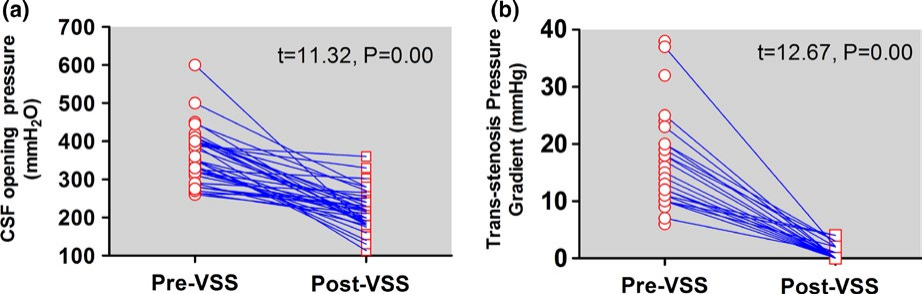
Changes of cerebrospinal fluid opening pressure and trans‐stenotic pressure gradient after treatment. (a) changes of trans‐stenotic pressure gradient; (b) changes of cerebrospinal fluid opening pressure

CSF opening pressure was documented in 83 patients before stenting, and the mean pressure was 339.07 mmH_2_O (ranging from 260–600). In the 53 patients for whom postoperative CSF opening pressure was available, it reduced to 221.08 ± 49.71 (ranging from 113–360). Moreover, opening pressure decreased after stenting in 52 patients, with only one patient experiencing a slight 10 mmH_2_O increase after stenting. The remaining patients did not receive lumbar punctures because of symptom improvement or lost to follow‐up, and no data were retrievable. The pre‐ and postpaired comparison of patients’ opening pressure shows a significant decrease after stenting (*t* = 11.32, *p* = 0.00) (Figure 1a). The average preoperative trans‐stenotic pressure gradient across 54 patients was 15.43 ± 7.08 (6–38) mmHg, which decreased to 0.81 (0–4) mmHg after stenting. These results indicate a significant change (*t* = 12.67, *p* = 0.00) (Figure 1b).

### 3.5 Associations between BMI and Trans‐stenotic pressure gradient, CSF opening pressure, and optical outcome

It is well‐acknowledged that IIH preferably affects overweight women and thus BMI may act as a risk factor of IIH. Therefore, we further analyzed the associations between patient BMI and intracranial pressure, and trans‐stenotic pressure gradient and subjective visual clinical outcome. The association between BMI categories (normal, overweight, and obese) and CSF opening pressure and trans‐stenotic pressure gradient was determined. Interestingly, we found that the normal weight patients presented slightly higher CSF opening pressure than the other two groups. However, they also had the most significant shifts after stenting, compared with the other two patient groups (*F* = 3.547, *p* = 0.037). A gradual increase of trans‐stenotic pressure gradient with elevated BMI was observed in patients with IIH both before and after stenting. For patients with normal BMI, no residual pressure gradient was indicated after treatment (Table 3). A negative correlation was observed between baseline BMI and a change in opening pressure before and after stenting, indicating that low BMIs benefit more from venous sinus stenting in suppressing high intracranial pressure.

**Table 3:** Association between BMI with CSF opening pressure and trans‐stenosis pressure gradient

BMI (kg/m^2^)	CSF opening pressure (mmH_2_O)			Trans‐stenosis pressure gradient (mmHg)		
	Preop	Postop	Change	Preop	Postop	Change
≤24.5	360.62 ± 87.69	236.25 ± 54.07	202.50 ± 99.14	14.22 ± 5.33	0.00 ± 0.00	14.0 ± 9.14
24.5~29.5	325.12 ± 64.87	214.29 ± 51.03	126.58 ± 75.5	15.45 ± 7.37	1.00 ± 1.51	14.8 ± 7.67
≥29.5	344.38 ± 62.39	231.00 ± 34.28	143.78 ± 82.73	16.33 ± 8.5	1.13 ± 1.36	15.8 ± 5.61
*F* value	1.837	1.025	**3.547**	0.212	1.268	0.104
*p* value	0.167	0.367	**0.037**	0.810	0.298	0.902

Note

Bold indicates the differences among the 3 groups are statistically significant.

In addition, the results demonstrated that the baseline BMI was not necessarily related to a poor optical prognosis of the patients after stenting (Fisher's exact = 0.270; *p* = 0.874). Besides, we noticed that all five patients who had vision loss were either overweight or obese. During the follow‐up, 48 patients were tracked for changes in body weight. Of those, 25 remained stable, 13 lost weight, and 10 gained. We then examined whether body weight change after stenting could markedly affect clinical outcomes. It was showed that postoperative body weight control or gain did not significantly alter the population outcomes (Fisher's exact = 3.81; *p* = 0.432) (Table 4).

**Table 4:** Association between baseline BMI and follow‐up weight changes with subjective visual outcome

BMI or weight change	Outcome (n)			Statistics
	Improved	Worsened	No change	
Baseline BMI (kg/m^2^)				Fisher’s exact value = 0.270; *p* = 0.874
≤24.5	11	0	2	
24.5~29.5	27	4	2	
≥29.5	8	1	0	
Follow‐up Weigh changes				Fisher’s exact value** **=** **3.81; *p* ** **=** **0.432
No change (*n* = 25)	19	4	1	
Gain weight (*n* = 10)	5	0	0	
Lose weight (*n* = 13)	11	0	0	

### 3.6 Preliminary analysis of the factors associated with optical outcome after stenting

In this study, we conducted a preliminary investigation of potential risk factors associated with the poor prognosis (worsened and no change) of ophthalmological symptoms using logistic regression analysis of stenting outcomes. The multivariate analysis showed that female gender, stent type, and papilledema and vision dysfunction were associated with an increased risk of poor prognosis. However, no significant differences were indicated in these variables (Table 5).

**Table 5:** Logistic analysis of the risk factors related ophthalmological outcome after stenting

Variables	B	Sig.	OR	OR 95%C.I.	
				Lower	Upper
Gender	0.524	0.818	1.690	0.019	147.749
Age	0.036	0.670	1.037	0.878	1.224
BMI	0.122	0.490	1.130	0.799	1.597
Clinical duration	−0.015	0.852	0.985	0.839	1.157
Trans‐stenosis pressure gradient prior stenting	0.031	0.690	0.970	0.834	1.127
Stent types	1.187	.202	3.277	0.529	20.309
Pre papilledema	0.702	0.672	2.017	0.078	52.141
Vision dysfunction	2.252	0.113	9.502	0.589	153.270
Pre LP	0.011	0.526	1.011	0.977	1.047

## 4 DISCUSSION

Venous sinus stenosis is a relatively recent discovery observed in approximately 90% of patients with IIH, and thus venous sinus stenting is an alternative treatment modality. In this study, we summarized the effect of venous sinus stenting on patients with IIH and stenosis and further evaluated potential factors that affect the efficiency of stenting.

It is considered that the obstructed venous outflow at venous stenosis will induce venous sinus hypertension and CSF reabsorption deficiency across the arachnoid villi. The elevated pressure derived from CSF compresses brain parenchyma and the compliant venous sinus system, in turn leading to increased intracranial hypertension which is responsible for the IIH symptoms and obstructed venous outflow (Esfahani et al., 2015; Lenck et al., 2017). Cerebral stents can effectively treat high‐frequency venous sinus stenosis and the resultant intracranial pressure (Patsalides et al., 2019). Higgins, Owler, Cousins, and Pickard (2002) firstly reported their experience in treating a case of IIH using venous sinus stenting in 2002, and reduced pressure gradient and symptomatic improvement were documented in this patient. During the past 20 years, venous sinus stenting has been gradually applied in clinical management of IIH, given the high technical and clinical success and low complication rates with stenting (Satti et al., 2015). Very recently, study by Shields et al. (2019) demonstrates that venous sinus stenting is relatively safe and efficacious in most patients if the pressure gradient across the transverse sigmoid junction was above 5 mm Hg. In our study, immediate resolution of the trans‐stenotic pressure gradient was confirmed in all patients. Most patients also had decreased CSF opening pressures, as estimated by lumbar puncture after stenting. In addition to the intracerebral pressure resolving effect, venous sinus stenting also alleviates IIH symptoms. A recent study documented that stenting improved or resolved papilledema in 88% of patients and improved or resolved headaches in 84% (Aguilar‐Pérez et al., 2017; Levitt et al., 2017). Several systemic reviews and meta‐analyses have supported the efficacy and safety of venous sinus stenting to treat IIH. Teleb et al. summarized 19 studies comprising 207 patients who underwent transverse sinus stenting and found that headaches resolved or improved in 81% of patients and that papilledema improved in 90% (172/189), with an overall symptom improvement rate of 87% (Teleb et al., 2013). Starke's review found 10 (5.4%) complications reported among 185 patients from a database covering 2002–2014, including a similar symptom improvement rate (Starke et al., 2015). In a more recent updated review, it is concluded from 29 articles comprising 410 patients that venous sinus stenting is associated with high technical success (99.5%), low rates of repeated procedure (10%) and low major complication rates (1.5%) (Saber, Lewis, Sadeghi, Rajah, & Narayanan, 2018). In our study, significant subjective ophthalmological improvement or recovery was reported in 84.5% of patients, which is consistent with these previous studies. In patients with poor outcomes, 4 (7.0%) had unchanged symptoms and 5 (8.8%) had permanent loss of vision. These patients mainly had severe vision dysfunction before stenting. In a meta‐analysis by Saber et al, clinical outcome, stent survival and stent‐adjacent stenosis rates in 473 patients from 24 studies undergoing venous sinus stenting were reviewed. It was noticed that stent‐adjacent stenosis rate following stenting reached 14%, which could be a cause of repeated surgery and poor prognosis (Leishangthem, Sirdeshpande, Dua, & Satti, 2018). Although risk factors and clinical presentation seemed to be homogeneous, individual prognoses were difficult to ascertain in IIH populations. Nevertheless, our results suggest that using stenting for early diagnosis and prevention of IIH is beneficial for resolution and improvement clinical symptoms.

Body weight is a well‐acknowledged risk factor that is experientially considered to be important for relieving symptoms of IIH. Even one study reported that as little as 6% reduction of the body weight was effective for relieving IIH symptoms (Wall et al., 2014). In our study, we found that the trans‐stenotic pressure gradient increased with escalating BMI prior to treatment and that baseline BMI was associated with the capacity of stenting to change intracerebral pressure and the trans‐stenotic pressure gradient. Compared to normal‐weight patients, overweight and obese patients experienced greater decreases in the trans‐stenotic pressure gradient. Raper et al. reported that degrees of improvement in the pressure gradient after venous sinus stenting was greatest among obese and morbidly obese patients with BMI > 30 kg/m^2^ (Raper et al., 2017).

We traced body weight changes of patients after treatment and analyzed whether losing weight improved their symptoms. No significant impact of weight change on visual outcome was detected during follow‐up. Chagot et al. have suggested that weight gain, rather than initial weight, is a leading factor of poor visual outcome in patients with IIH using first‐line treatment (Chagot et al., 2017). We propose that venous sinus stenting may be a powerful covariate that affects the outcome. Recently, a program has been launched to evaluate the influence of weight loss on IIH symptoms and intracranial pressure (Ottridge et al., 2017). Our study is limited by its retrospective nature and lost to follow‐up in spite of that the follow‐up missing patients had comparable age, gender, BMI, initial symptoms, and pressures with those who were followed‐up. These patients came from all over the country and had high population mobility with someone changing contact information after discharge. Although the data revealed an association between BMI and intracranial pressure and provided some clues about potential risk factors for vision outcomes after stenting, no significant statistical difference were shown. Thus, the efficiency of this treatment should be validated in large perspective cohort, preferably in a multicenter setting. Besides, as the venous sinus stenting procedure has not yet been evaluated in randomized controlled or sham procedure trials, prospective studies are needed to address the clinical efficiency or long‐term outcomes of stenting and possible mechanisms of IIH.

## 5 CONCLUSION

Venous sinus stenting showed excellent efficiency in resolving vision impairment and suppressing CSF opening pressure and the trans‐stenotic pressure gradient caused by venous sinus stenosis. Baseline BMI was associated with the pre‐ and postoperative trans‐stenotic pressure gradient and with changes in CSF opening pressure. However, no association between baseline BMI or body weight changes during follow‐up and visual outcomes after stenting.

## CONFLICT OF INTEREST

All authors declare that they have no conflicts of interest.
